# Canadian nurses’ perspectives on challenges to antimicrobial stewardship in long-term care homes

**DOI:** 10.14745/ccdr.v52i04a06

**Published:** 2026-04-30

**Authors:** Alexander Varsaneux, Kathleen Qu, Jorida Cila, Gabrielle Brankston, Klajdi Puka, Tyler Good, Barbara Catt, Shaghig Reynolds, Aboubakar Mounchili, Denise Gravel-Tropper

**Affiliations:** 1 Centre for Communicable Diseases and Infection Control, Public Health Agency of Canada, Ottawa, ON; 2 Surveillance, Integrated Insights and Risk Assessment, Public Health Agency of Canada, Ottawa, ON; 3 Impact Canada, Privy Council Office, Ottawa, ON

**Keywords:** antimicrobial stewardship, antimicrobial resistance, long-term care, nurses, survey, infection prevention and control

## Abstract

**Background:**

The World Health Organization recognizes antimicrobial resistance (AMR) as a top global health threat. Antimicrobial resistance-related deaths in Canada are projected to exceed 13,500 annually by 2050. In long-term care homes (LTCH), 40% to 75% of antimicrobial prescriptions are inappropriate or unnecessary. Nurses are well-positioned to lead in antimicrobial stewardship (AMS) initiatives; however, it is unclear what challenges LTCH nurses face in participating in AMS activities. This study aims to provide insights into barriers and facilitators influencing nurses’ engagement in AMS within Canadian LTCHs.

**Methods:**

A survey was developed using literature and an AMS working group assessing AMS knowledge (10 questions), confidence, barriers and facilitators. The survey targeted nurses working in Canadian LTCHs and was administered through Qualtrics online from January 20, 2023 to April 10, 2023 by distribution partners to obtain a convenience sample. Open-coding thematic analysis was used to describe quantitative data and analyze qualitative responses.

**Results:**

A total of 346 complete responses were recorded. The mean knowledge score was 71% (standard deviation [SD]=15%). Most respondents perceived infection prevention and control measures, and monitoring changes in the health of residents to be part of the duties of nurses within AMS. However, making recommendations about antimicrobials was the least cited nursing responsibility. This suggests a lack of clarity around the role of nurses in AMS. Barriers to AMS activities included pressure to treat and lack of meaningful interprofessional communication, while AMS education and senior management support were drivers for AMS engagement.

**Conclusion:**

Education, senior-level support, and formal recognition of nurses in AMS programming represent key facilitators to effectively engage LTCH nurses in AMS best practices.

## Introduction

Antimicrobial resistance (AMR) is recognized as a public health risk globally ((1)) and in Canada ((2)). According to the Council of Canadian Academies by 2050, deaths in Canada attributable to AMR is predicted to reach over 13,500 per year ((3)). In long-term care home (LTCH) settings, 40% to 75% of antimicrobial prescriptions are inappropriate or unnecessary ((4–7)). Antimicrobial stewardship (AMS) programs use a systematic approach to improve judicious antimicrobial use (AMU) and promote behavioural changes, commonly focusing on the beliefs and motivations of prescribers ((8,9)).

Long-term care home physicians often oversee over 50 residents ((10)) and, in turn, rely on nurses, who interact frequently with residents and their caregivers, to monitor and contribute to residents’ care plans ((11)). Recognizing the potential for nurses to lead AMS, the Canadian Nurses Association (CNA) Antimicrobial Stewardship Competencies: A Pan-Canadian Framework for Nurses identified seven AMS domains to effectively promote and participate in AMS in nurse practice settings ((12)). While nurses have been identified as important and successful AMS stewards, and some research focuses on the roles, perspectives, and challenges for nurses around AMR and AMS ((13–15)), there is no clear literature focusing on the Canadian LTCH context specifically targeting nurses working in these settings. This study aims to describe the current landscape, based on the perspectives of nurses in Canadian LTCH settings, to identify the barriers and facilitators that influence their engagement in AMS, thereby informing future initiatives and enhancing their contributions to AMS activities.

## Methods

### Survey design

Survey development involved an informal literature review on AMS initiatives that include nursing staff and was overseen by a working group of infectious disease physicians, behavioural scientists and infection prevention and control (IPC) experts. For face validity, appropriate length and clarity, the survey was piloted by four LTCH nurses.

The anonymous 15 minute online survey (**Appendix**, Table S1) ran from January 20, 2023 to April 10, 2023, targeting Canadian LTCH nurses via convenience sampling through distribution partners (Appendix, Table S2). Informed consent was obtained, with no incentives, and data privacy followed the *Canadian Privacy Act*.

The survey consisted of four sections: participant demographics, knowledge, perspectives, and barriers and facilitators. Two open-ended questions were included for additional comments.

### Demographics

Demographics included educational background, work experience, certifications and previous AMS/AMR training.

### Knowledge

Eighteen knowledge questions were adapted from the literature to determine each respondent’s knowledge of AMS practices ((16–20)). Topics included defining relevant concepts (AMR, AMS, IPC, judicious AMU), and identification and testing for respiratory tract infections (RTIs), and urinary tract infections (UTIs) ((16–25)) (Appendix, Table S1). Ten of 18 questions were randomly displayed to respondents.

### Perspectives

Respondents were asked about their perspectives on AMU ((8)), influence on prescribing antimicrobials, responsibilities related to AMS, and their self-reported behaviours around AMR ((22,24)). Confidence related to performing AMS-related tasks was assessed using Likert scales ((25)) (Appendix, Table S1).

### Barriers and facilitators

Twelve barrier indicators and ten facilitator indicators for AMS activities were identified through literature ((26)) and examined using Likert scales.

Descriptive analysis was conducted using R software ((27)) and Microsoft Excel ((28)), as well as open-ended thematic coding to analyze qualitative responses. Due to a low response rate, no sub-group analyses were conducted.

## Results

The survey was completed by 346 LTCH nurses (326 female, 94%), and 106 (31%) of the respondents were 45–54 years old) (**Table 1**). One quarter (92/346, 27%) of respondents were from Alberta, followed by Manitoba (79/346, 23%), New Brunswick (66/346, 19%), and Ontario (61/346, 18%). Over half (195/346, 56%) of respondents had more than 10 years of experience working in LTCHs, 57% worked full-time, and most were registered nurses (64%). Respondents also included eleven (3%) nurses who were directors of care or in manager positions.

**Table 1:** Demographics

Respondent characteristics (n=346)	Number (%)
**Age**	
<35	47 (14%)
35–44	98 (28%)
45–54	106 (31%)
55–64	82 (24%)
65+	13 (4%)
**Gender**	
Woman	326 (94%)
Man	16 (4.6%)
Other/undeclared	2 (0.6%)
Prefer not to say	1 (0.3%)
No response	1 (0.3%)
**Experience**	
>10 years LTCH nursing	195 (56%)
>10 years total nursing experience	259 (75%)
**Employment type**	
Full-time	197 (57%)
Part-time	112 (32%)
Casual	30 (9%)
**Nursing title**	
Nurse practitioner (NP)	4 (1%)
Registered nurse (RN)	217 (61%)
Licensed practical nurse (LPN)/Registered practical nurse (RPN)	101 (30%)
Other	15 (4%)
**Certification**	
Gerontology	151 (44%)
Infection prevention and control	64 (19%)
Wound, ostomy, and continence	33 (10%)
Hospice	19 (6%)
Psychiatric	18 (5%)
Other	26 (8%)
None	117 (34%)
**Type of LTCH**	
Public	166 (48%)
Private non-profit/for-profit	139 (40%)
Don’t know/unsure	41 (12%)
**Province of practice^a^**	
Alberta	92 (27%)
Saskatchewan	20 (6%)
Manitoba	79 (23%)
Ontario	61 (18%)
New Brunswick	66 (19%)
Nova Scotia	25 (7%)
Prince Edward Island	3 (1%)

Abbreviation: LTCH, long-term care home

^a^ No responses were received from Québec, British Columbia, Newfoundland and the territories

### Antimicrobial stewardship knowledge

**Figure 1:**
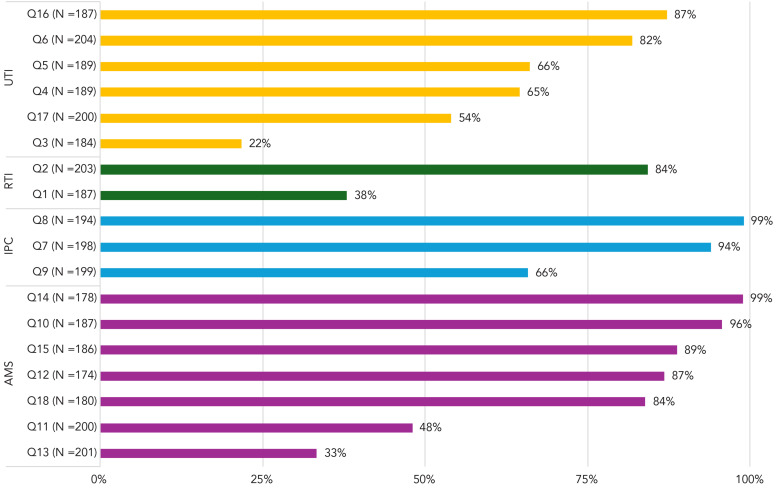
Knowledge questions Abbreviations: AMS, antimicrobial stewardship; IPC, infection prevention and control; RTI, respiratory tract infection; UTI, urinary tract infection

The mean score for the knowledge questions was 71% (7/10) (standard deviation [SD]=15%) with a range of 1/10 to 10/10 (**Figure 1**). The highest scores were reported for IPC questions (86%, SD=28%), followed by AMS knowledge questions (75%, SD=24%). The lowest scores were for RTI-related questions (62%, SD=43%). Forty-two percent (146/346) of respondents indicated awareness of an AMR or AMS policy at their LTCH. Thirty-three percent (114/346) of respondents reported not receiving AMR training in the past twelve months.

### Perspectives

Eighty-one percent (281/346) of respondents reported that AMU is a problem in Canada, however, only 41% (143/346) indicated that antimicrobial overuse is a problem at their LTCH. Most respondents felt physicians (335/346, 97%) and pharmacists (308/346, 89%) were highly responsible for AMS, while 87% felt nurses were highly responsible for AMS activities.

Most respondents perceived the following stewardship activities to be part of their responsibilities: IPC measures (340/346, 98%), monitoring changes in resident health status, with certain signs and symptoms that would indicate the need for testing and potential treatment (337/346, 97%), and monitoring side effects of medication (318/346, 92%). Providing recommendations on appropriate dosage and duration (148/346, 43%), discontinuation of antimicrobials (134/346, 39%), and use of narrow-spectrum antimicrobials (127/346, 37%) were activities least identified to be responsibilities of nurses. Over half of respondents (199/346, 58%) reported that when testing for UTIs, they always or often consider AMR. In practice, 53% (184/346) report administering or overseeing two or fewer urine cultures per month. Most respondents (282/346, 82%) felt at least somewhat comfortable raising concerns about antimicrobials to physicians, and 75% of respondents believe their interdisciplinary communication with physicians and pharmacists can influence antimicrobial prescribing. However, 46% (159/346) of respondents believed that they sometimes or often gave antimicrobials that they thought were inappropriate.

Respondents had high overall confidence in performing AMS tasks (average 4.1/5, SD=0.6). Respondents felt least confident identifying an incorrectly prescribed antimicrobial class (3.3/5) and felt most confident recognizing signs and symptoms of UTI in residents (4.5/5). High confidence was not statistically significantly correlated with knowledge score.

### Barrier and facilitator indicators

**Figure 2:**
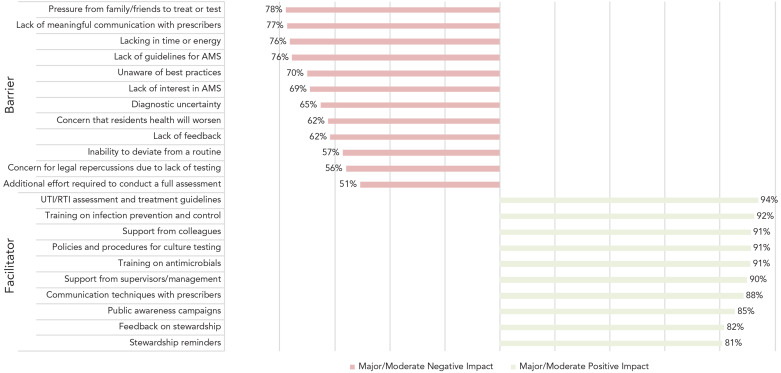
Barriers and facilitators Abbreviations: AMS, antimicrobial stewardship; RTI, respiratory tract infection; UTI, urinary tract infection

Responses ranged widely on how each barrier affected nurses’ ability to practice AMS at their LTCH (**Figure 2**). The most prevalent barriers were pressure from a resident’s family and friends to treat or test (269/346, 78%), lack of meaningful communication with prescribers (268/346, 77%), lack of time or energy (264/346, 76%), and lack of guidelines for AMS in the LTCH setting (261/346, 76%). The least cited barrier was having to conduct a full assessment, making antibiotics the easiest course of action (176/346, 51%).

For facilitators, UTI or RTI assessment and treatment guidelines (324/346, 94%) were perceived to positively influence the practice of AMS among nurses, followed by IPC training (320/346, 92%), policies and procedures for specimen testing (315/346, 91%), and support from colleagues (315/346, 91%). The lowest response was for stewardship reminders, such as AMR posters in the halls (280/346, 81%). It was unknown whether respondents had access to all facilitators, however, all facilitators were perceived to be beneficial in promoting AMS practices. There were no significant differences in facilitators or barrier ranking when comparing nurses with over 10 years and less than 10 years of experience in LTCHs.

### Qualitative analysis

Of the 346 respondents, 159 (46%) provided open-ended responses. Key facilitators included education, workplace guidelines, advocacy, and provincial/national AMS guidelines. Responses highlighted successful implementation, adequate staffing, and management endorsement and participation. One noted the influence of culture and collaboration: “It is an antibiotic first culture. We need to focus on other non-pharmaceutical interventions to... maintain health and prevent infection are the key, the challenge is to have adequate staffing to achieve this. This is a systemic problem and requires the financial support of the government and the local leadership team.”

Respondents noted that everyone had a role in AMS and desired accessible training and inclusion of other healthcare staff and residents and their caregivers in AMS education. Improved interdisciplinary communication, collaboration and building a preventive culture were needed. Workload, staff shortages, and staff burnout were persistent barriers.

## Discussion

There is growing international interest in implementing and evaluating AMS in community settings ((29)). Internationally, the focus has been on nursing home staff (which often includes less clinically complex residents), however, LTCH nurses have not been well-studied in the context of AMS. It is necessary to understand the perspectives and knowledge of LTCH nurses to understand barriers and facilitators to AMS engagement.

Respondents felt highly responsible for many AMS activities. Nearly all respondents identified AMR as an issue and believe they can contribute positively to AMS at their workplace. Respondents performed best on IPC and AMS knowledge questions, suggesting familiarity with AMS practices. The widely ranging overall scores suggest variations in knowledge of AMS, including lack of AMS guidelines, lack of awareness of best practices, insufficient training, and low awareness of existing AMS policies. Furthermore, qualitative responses supported tailored education, AMS-specific training and specific guidelines to promote the involvement of nurses.

Respondents were confident in their abilities to perform AMS tasks, specifically those related to education on AMU, recognizing signs of infection, and performing tests in line with IPC practices. However, confidence did not correlate with better knowledge scores as noted among other healthcare professionals ((30)). High confidence reinforced the suggestion that many nurses are familiar with AMS tasks and frequently perform them. Formally recognizing their roles and supplying the appropriate training and support can address gaps in stewardship knowledge ((9,15,29)).

Interprofessional communication is critical for combatting AMR in LTCHs ((29,31–33). While most nurses felt comfortable raising concerns to physicians about antimicrobial therapy, almost half felt they may be administering inappropriate antimicrobials. A lack of meaningful communication suggests nurses’ concerns are not taken into consideration by other health professionals. The framework (published after survey distribution) identifies timely communication and discussion on antimicrobial therapy as core competencies ((12)), however, survey findings suggest current lines of communication may not be sufficient for effective interprofessional collaboration and thus should be prioritized.

Over three quarters of respondents identified seven of the eleven stewardship activities to be part of a nurse’s responsibility in LTCHs, confirming that nurses already take on stewardship roles ((34–37)). However, less than half perceived recommendations on antimicrobial therapy as their responsibility, highlighting a discrepancy in AMS involvement for these activities. While most LTCH physicians are the prescribers, LTCH nurses are the most present regulated healthcare professionals and significantly influence residents’ care plans, including decision-support for antimicrobial therapy ((11,13)). Notably, the framework and AMS best practices highlight two competencies: appropriate use of antimicrobial agents and interprofessional collaborative practice, which can involve recommendations from nurses on antimicrobial therapy ((12,13)). Potential reasoning for the gap in nurses’ perspectives include unclear AMS responsibilities, a lack of confidence in their AMS knowledge, and a lack of interprofessional relationships that foster collaborative AMS discussions. The framework also promotes clear role definition, interprofessional collaboration, and ongoing education ((12)). Formal inclusion of nurses in AMS activities acknowledges their significant contributions, strengthens their role in antimicrobial decision-making, and empowers them to actively participate and lead in AMS initiatives within their organizations ((38)).

All listed facilitators were well-received by respondents, indicating implementing any of them could be beneficial (Figure 2). Respondents specifically mentioned buy in from senior management and other healthcare professionals would be extremely beneficial to their AMS activities, partly because they perceive that they are working in an “antimicrobials-first culture” with little senior leadership that encourages AMS. The listed facilitators can be used to identify optimal strategies for incorporating nurses in AMS activities in their local context. The listed barriers had greater variability in perceived negative effect, suggesting they may be more context specific.

### Limitations

This first national AMS survey of Canadian LTCH nurses was timely amid growing interest in disease prevention and AMS. Use of open-ended responses enabled respondents to provide clarity and voice concerns. The results relied on convenience sampling, introducing selection bias. Survey distribution in a post-COVID-19 pandemic context may have reduced response rate, representativeness and generalizability, as only those with strong interest in AMS may have participated.

## Conclusion

Canadian nurses in LTCHs perceive AMS as an important part of their responsibilities, and many already include AMS activities in their routines. The AMS knowledge and responsibilities of nurses vary. Key challenges include interprofessional communication, inconsistent AMS roles, and an antimicrobials-first culture. Leading facilitators include having AMS guidelines, tailored education, senior-level endorsement, and formalized AMS roles. Future representative sample studies should identify core AMS competencies relevant to LTCH nurses. Local nuances can be identified through replicating this study locally in consideration of local or province-specific AMS policies and guidelines.

## Appendix

Supplemental material is available upon request to the author: amrtf-gtram@phac-aspc.gc.ca

Table S1: Survey questions

Table S2: Survey recruitment and distribution process

## References

[1] World Health Organization. Antimicrobial resistance. Geneva, CH: WHO; 2023. [Accessed 2024 Oct 29]. https://www.who.int/news-room/fact-sheets/detail/antimicrobial-resistance

[2] Public Health Agency of Canada. Canadian Antimicrobial Resistance Surveillance System Report. Ottawa, ON: PHAC; 2021. [Accessed 2024 Oct 29]. https://www.canada.ca/content/dam/phac-aspc/documents/services/publications/drugs-health-products/canadian-antimicrobial-resistance-surveillance-system-report-2021/canadian-antimicrobial-resistance-surveillance-system-report-2021.pdf

[3] Council of Canadian Academies. When Antibiotics Fail. Ottawa, ON: CCA; 2019. https://cca-reports.ca/reports/the-potential-socio-economic-impacts-of-antimicrobial-resistance-in-canada/

[4] Penney CC, Boyd SE, Mansfield A, Dalton J, O’Keefe J, Daley PK. Antimicrobial use and suitability in long-term care facilities: a retrospective cross-sectional study. J Assoc Med Microbiol Infect Dis Can 2018;3(4):209–16. https://doi.org/10.3138/jammi.2018-0021

[5] Daneman N, Bronskill SE, Gruneir A, Newman AM, Fischer HD, Rochon PA, Anderson GM, Bell CM. Variability in antibiotic use across nursing homes and the risk of antibiotic-related adverse outcomes for individual residents. JAMA Intern Med 2015;175(8):1331–9. https://doi.org/10.1001/jamainternmed.2015.2770 26121537

[6] Thornley T, Ashiru-Oredope D, Normington A, Beech E, Howard P. Antibiotic prescribing for residents in long-term-care facilities across the UK. J Antimicrob Chemother 2019;74(5):1447–51. https://doi.org/10.1093/jac/dkz008 30698718 PMC6477989

[7] Schwartz KL, Langford BJ, Daneman N, Chen B, Brown KA, McIsaac W, Tu K, Candido E, Johnstone J, Leung V, Hwee J, Silverman M, Wu JH, Garber G. Unnecessary antibiotic prescribing in a Canadian primary care setting: a descriptive analysis using routinely collected electronic medical record data. CMAJ Open 2020;8(2):E360–9. https://doi.org/10.9778/cmajo.20190175 32381687 PMC7207032

[8] Abbo L, Smith L, Pereyra M, Wyckoff M, Hooton TM. Nurse Practitioners’ attitudes, perceptions, and knowledge about antimicrobial stewardship. J Nurse Pract 2012;8(5):370–6. https://doi.org/10.1016/j.nurpra.2012.01.023

[9] Davey K, Aveyard H. Nurses’ perceptions of their role in antimicrobial stewardship within the hospital environment. An integrative literature review. J Clin Nurs 2022;31(21-22):3011–20. https://doi.org/10.1111/jocn.16204 35092116 PMC9787640

[10] Lam JM, Anderson GM, Austin PC, Bronskill SE. Family physicians providing regular care to residents in Ontario long-term care homes: characteristics and practice patterns. Can Fam Physician 2012;58(11):1241–8. 23152465 PMC3498022

[11] Armstrong P, Armstrong H, Choiniere J, Lowndes R. RNs in Long-Term Care: A Portrait. 2019. Report prepared for the Ontario Nurses Association. ONA.

[12] Canadian Nurses Association. Antimicrobial stewardship competencies: a pan-Canadian framework for nurses. Ottawa, ON: CNA; 2023. https://cna.informz.ca/cna/pages/AMS_framework_landing_page

[13] American Nurses Association and Center for Disease Control and Prevention. Redefining the Antibiotic Stewardship Team: Recommendations from the American Nurses Association/Centers for Disease Control and Prevention Workgroup on the Role of Registered Nurses in Hospital Antibiotic Stewardship Practices. Silver Spring, MD: ANA/CDC; 2017. https://www.cdc.gov/antibiotic-use/healthcare/pdfs/ANA-CDC-whitepaper.pdf

[14] Olans RN, Olans RD, DeMaria A Jr. The Critical Role of the Staff Nurse in Antimicrobial Stewardship--Unrecognized, but Already There. Clin Infect Dis 2016;62(1):84–9. https://doi.org/10.1093/cid/civ697 26265496

[15] Nie H, Yue L, Peng H, Zhou J, Li B, Cao Z. Nurses’ engagement in antimicrobial stewardship and its influencing factors: A cross-sectional study. Int J Nurs Sci 2023;11(1):91–8. https://doi.org/10.1016/j.ijnss.2023.12.002 38352296 PMC10859584

[16] Brink AJ, Van Wyk J, Moodley VM, Corcoran C, Ekermans P, Nutt L, Boyles T, Perovic O, Feldman C, Richards G, Mendelson M. The role of appropriate diagnostic testing in acute respiratory tract infections: an antibiotic stewardship strategy to minimise diagnostic uncertainty in primary care. S Afr Med J 2016;106(6):30–7. 27245715 10.7196/SAMJ.2016.v106i6.10857

[17] Jump RL, Heath B, Crnich CJ, Moehring R, Schmader KE, Olds D, Higgins PA. Knowledge, beliefs, and confidence regarding infections and antimicrobial stewardship: a survey of Veterans Affairs providers who care for older adults. Am J Infect Control 2015;43(3):298–300. https://doi.org/10.1016/j.ajic.2014.11.017 25728158 PMC4347997

[18] Loeb M, Bentley DW, Bradley S, Crossley K, Garibaldi R, Gantz N, McGeer A, Muder RR, Mylotte J, Nicolle LE, Nurse B, Paton S, Simor AE, Smith P, Strausbaugh L. Development of minimum criteria for the initiation of antibiotics in residents of long-term-care facilities: results of a consensus conference. Infect Control Hosp Epidemiol 2001;22(2):120–4. https://doi.org/10.1086/501875 11232875

[19] Wretman CJ, Boynton MH, Preisser JS, Zimmerman S, Kistler CE. Patient-level information underlying overdiagnosis of urinary tract infections in nursing homes: A discrete choice experiment. Infect Control Hosp Epidemiol 2023;44(7):1151–4. https://doi.org/10.1017/ice.2022.171 36073169

[20] Choosing Wisely Canada. Allergy & Clinical Immunology Seven Things Clinicians and Patients Should Question by Canadian Society of Allergy and Clinical Immunology. 2023. https://choosingwiselycanada.org/recommendations/

[21] World Health Organization. Antimicrobial stewardship interventions: a practical guide. Geneva, CH: WHO; 2021. https://www.who.int/europe/publications/i/item/9789289056267

[22] Merrill K, Hanson SF, Sumner S, Vento T, Veillette J, Webb B. Antimicrobial stewardship: staff nurse knowledge and attitudes. Am J Infect Control 2019;47(10):1219–24. https://doi.org/10.1016/j.ajic.2019.03.022 31128981

[23] Alberta Health Services. Managing Coughs, Colds, Sneezes & Germs Guide to Wise Use of Antibiotics. Edmonton, AB: AHS; 2022. www.dobugsneeddrugs.org

[24] Wilson BM, Shick S, Carter RR, Heath B, Higgins PA, Sychla B, Olds DM, Jump RL. An online course improves nurses’ awareness of their role as antimicrobial stewards in nursing homes. Am J Infect Control 2017;45(5):466–70. https://doi.org/10.1016/j.ajic.2017.01.002 28189411 PMC5410397

[25] Monsees E, Lee B, Wirtz A, Goldman J. Implementation of a nurse-driven antibiotic engagement tool in 3 hospitals. Am J Infect Control 2020;48(12):1415–21. https://doi.org/10.1016/j.ajic.2020.07.002 32645472

[26] McArthur C, Bai Y, Hewston P, Giangregorio L, Straus S, Papaioannou A. Barriers and facilitators to implementing evidence-based guidelines in long-term care: a qualitative evidence synthesis. Implement Sci 2021;16(1):70. https://doi.org/10.1186/s13012-021-01140-0 34243789 PMC8267230

[27] R Core Team. R: A Language and Environment for Statistical Computing. Computer software; Version 4.4.1. R Foundation for Statistical Computing. Vienna, AUT: R Core Team; 2024. https://www.R-project.org/

[28] Excel M. Computer software; Version 2408 Build 16.0.17928.20114. Microsoft Corporation. 2024.

[29] Belan M, Thilly N, Pulcini C. Antimicrobial stewardship programmes in nursing homes: a systematic review and inventory of tools. J Antimicrob Chemother 2020;75(6):1390–7. https://doi.org/10.1093/jac/dkaa013 32108883

[30] Gharbi M, Moore LS, Castro-Sánchez E, Spanoudaki E, Grady C, Holmes AH, Drumright LN. A needs assessment study for optimising prescribing practice in secondary care junior doctors: the Antibiotic Prescribing Education among Doctors (APED). BMC Infect Dis 2016;16(1):456. https://doi.org/10.1186/s12879-016-1800-z 27576784 PMC5006515

[31] Monsees EA, Tamma PD, Cosgrove SE, Miller MA, Fabre V. Integrating bedside nurses into antibiotic stewardship: A practical approach. Infect Control Hosp Epidemiol 2019;40(5):579–84. https://doi.org/10.1017/ice.2018.36230786944

[32] Chambers A, MacFarlane S, Zvonar R, Evans G, Moore JE, Langford BJ, Augustin A, Cooper S, Quirk J, McCreight L, Garber G. A recipe for antimicrobial stewardship success: using intervention mapping to develop a program to reduce antibiotic overuse in long-term care. Infect Control Hosp Epidemiol 2019;40(1):24–31. https://doi.org/10.1017/ice.2018.281 30394258

[33] Fleming A, Bradley C, Cullinan S, Byrne S. Antibiotic prescribing in long-term care facilities: a meta-synthesis of qualitative research. Drugs Aging 2015;32(4):295–303. https://doi.org/10.1007/s40266-015-0252-2 25832969 PMC4412731

[34] Monsees E, Popejoy L, Jackson MA, Lee B, Goldman J. Integrating staff nurses in antibiotic stewardship: opportunities and barriers. Am J Infect Control 2018;46(7):737–42. https://doi.org/10.1016/j.ajic.2018.03.028 29729830

[35] Fisher CC, Cox VC, Gorman SK, Lesko N, Holdsworth K, Delaney N, McKenna C. A theory-informed assessment of the barriers and facilitators to nurse-driven antimicrobial stewardship. Am J Infect Control 2018;46(12):1365–9. https://doi.org/10.1016/j.ajic.2018.05.020 30077436

[36] Scales K, Zimmerman S, Reed D, Beeber AS, Kistler CE, Preisser JS, Weiner BJ, Ward K, Fann A, Sloane PD. Nurse and Medical Provider Perspectives on Antibiotic Stewardship in Nursing Homes. J Am Geriatr Soc 2017;65(1):165–71. https://doi.org/10.1111/jgs.14504 28111755

[37] Giblin TB, Sinkowitz-Cochran RL, Harris PL, Jacobs S, Liberatore K, Palfreyman MA, Harrison EI, Cardo DM; CDC Campaign to Prevent Antimicrobial Resistance Team. Clinicians’ perceptions of the problem of antimicrobial resistance in health care facilities. Arch Intern Med 2004;164(15):1662–8. https://doi.org/10.1001/archinte.164.15.1662 15302636

[38] Gotterson F, Buising K, Manias E. Nurse role and contribution to antimicrobial stewardship: an integrative review. Int J Nurs Stud 2021;117:103787. https://doi.org/10.1016/j.ijnurstu.2020.103787 33647845

